# Diagnostic performance of intraoperative urine dipstick testing during ureteroscopy: association with culture positivity and severe infection

**DOI:** 10.1007/s00240-026-02020-2

**Published:** 2026-06-13

**Authors:** Carlos González González, Pietro Scilipoti, Federico Zorzi, Nicola Nannola, Marie Chicaud, Frédéric Panthier, Olivier Traxer

**Affiliations:** 1 Endolase Lab, PIMM Lab Arts et Métiers ParisTech, GRC n°20-Sorbonne Université, Paris, 75020 France; 2 Service d’Urologie, Assistance-Publique Hôpitaux de Paris, Hôpital Tenon, Sorbonne Université, Paris, 75020 France; 3 PIMM, UMR 8006 CNRS-Arts et Métiers ParisTech, 151 bd de l’Hôpital, Paris, F-75013 France; 4 Department of Experimental Oncology, Unit of Urology, URI, IRCCS Ospedale San Raffaele, Milan, Italy; 5 Vita-Salute San Raffaele University, Milan, Italy; 6 Section of Endourology, European Association of Urology, Arnhem, Netherlands; 7 Endourology Technology Section of European Association of Urology (EAU), Arnhem, The Netherlands; 8 Progressive Endourological Association for Research and Leading Solutions (PEARLS), Paris, France

**Keywords:** Ureteroscopy, Urine dipstick, Diagnostic performance, Culture positivity, Postoperative infection, Sensitivity and specificity

## Abstract

Ureteroscopy (URS) is a widely performed procedure for urinary stone disease, yet infectious complications, including sepsis, remain a concern despite negative preoperative urine cultures. Intraoperative urine dipstick testing offers a rapid point-of-care assessment, but its diagnostic performance for predicting culture positivity and severe infection at different sampling sites during URS remains unclear. In this retrospective, single-center study, 238 patients undergoing URS were included. Paired urine dipstick and culture samples were obtained from bladder urine, intraoperative pelvic urine, and post-laser pelvic urine. Dipstick positivity was defined using two criteria: leukocytes and/or nitrites (OR definition) and leukocytes and nitrites (AND definition). Sensitivity, specificity, positive predictive value (PPV), and negative predictive value (NPV) with 95% confidence intervals were calculated for predicting culture positivity. Receiver operating characteristic (ROC) curve analysis was performed, including a composite dipstick score (0–2). Severe postoperative infection was assessed as a secondary exploratory outcome. Culture positivity ranged from 18.4% (bladder) to 22.9% (intraoperative pelvic urine). Using the OR definition, pooled sensitivity was 80.5% and specificity 47.6%, whereas the AND definition yielded lower sensitivity (43.8%) and higher specificity (86.4%). Diagnostic performance varied by sampling site, with intraoperative pelvic urine demonstrating the most balanced metrics. ROC analysis showed moderate discriminative performance, with the highest AUC observed for pelvic urine sampled prior to laser fragmentation (AUC 0.723, 95% CI 0.646–0.801). For severe infection (4.2%), dipstick testing demonstrated low sensitivity (30%), and this analysis was considered exploratory. Intraoperative urine dipstick testing demonstrates moderate diagnostic performance for predicting culture positivity during URS, with results influenced by sampling site and positivity definition. Pre-laser pelvic sampling provides the most informative results. A negative intraoperative dipstick, particularly from pre-laser pelvic urine, may help identify patients at lower likelihood of culture positivity; however, the test remains insufficient to guide antimicrobial or postoperative decision-making in isolation. Further prospective studies are required to validate its role in perioperative risk stratification.

**Supplementary Information:**

The online version contains supplementary material available at 10.1007/s00240-026-02020-2.

## Introduction

Ureteroscopy (URS) is a widely performed minimally invasive procedure for the management of urinary stone disease, with millions of cases annually worldwide [1]. Despite its efficacy, infectious complications, such as urinary tract infections and sepsis, remain a significant concern, contributing to increased morbidity, prolonged hospital stays, and healthcare costs [2]. Notably, severe infections can occur even in patients with negative preoperative urine cultures, highlighting the limitations of standard preoperative screening [3].

Intraoperative renal pelvic urine and/or stone cultures may provide additional microbiological information beyond preoperative bladder cultures and have been shown to better predict postoperative infectious complications [4]. Urine dipstick testing, which detects leukocytes and nitrites as markers of inflammation and bacterial activity, provides a rapid and inexpensive point-of-care diagnostic tool [5]. While urine dipstick testing is widely used in the outpatient setting, its diagnostic accuracy in predicting culture-confirmed infection from intraoperative upper tract urine samples during ureteroscopy remains insufficiently defined.

To date, no study has systematically evaluated the diagnostic performance of urine dipstick testing at multiple timepoints during URS, the impact of different positivity definitions (e.g., leukocytes and/or nitrites versus both), or its association with severe postoperative infections such as sepsis or septic shock.

We aimed to evaluate the diagnostic performance of intraoperative urine dipstick testing during ureteroscopy for predicting culture positivity and severe postoperative infection.

## Methods

### Study design and participants

This retrospective single-center cohort study was conducted at Hôpital Tenon (Paris, France), a tertiary referral endourology center. The study included consecutive patients treated with flexible ureterorenoscopy by a single surgeon (OT) and was conducted in accordance with national and institutional research regulations.

All patients were systematically screened preoperatively with urine culture and dipstick analysis performed on mid-stream bladder urine samples collected approximately 10 days prior to the scheduled surgery date. Intraoperatively, immediately upon accessing the renal pelvis, a pelvic urine sample was obtained for both urine culture and dipstick analysis. Finally, at the conclusion of the procedure—following laser lithotripsy if performed—an additional pelvic urine sample was collected for the same analyses. All samples were processed according to standard laboratory protocols, with urine cultures considered positive based on microbiologically confirmed bacterial growth (ECBU). Dipstick testing evaluated the presence of leukocytes and nitrites. Paired dipstick and culture results were analyzed for diagnostic performance, as detailed in the statistical analysis section.

This study was conducted in accordance with the Declaration of Helsinki and approved by the institutional ethics committee of Hôpital Tenon (Assistance Publique–Hôpitaux de Paris). Due to the retrospective nature of the study, the requirement for informed consent was waived by the ethics committee.

Eligible participants were adult patients (≥ 18 years) who underwent FURS for renal and/or ureteral calculi between June 2023 and October 2025. Patients were identified through prospectively maintained institutional surgical databases at our center. All patients had complete postoperative follow-up within the study period, and outcome data were verified prior to final analysis. No patients were lost to follow-up for the primary or secondary endpoints.

A total of 238 consecutive eligible patients were identified. (Table 1).

**Table 1:** Baseline characteristics of the study population

Characteristic	*N* = 238
Age, years	52 ± 19
Gender	
Female	102 (43%)
Male	136 (57%)
Body mass index, kg/m²	25.9 ± 5.8
Diabetes mellitus	32 (13%)
Hypertension	75 (32%)
Urine dipstick positivity — Intraoperative Bladder	151 (63%)
Urine dipstick positivity — Intraoperative Pre-Laser Pelvic	132 (55%)
Urine dipstick positivity — Intraoperative Post-Laser Pelvic	93 (39%)
Urine culture positivity — Pre-Laser Pelvic Urine Culture	48 (23%)
Severe infection	10 (4.2%)

^a^ Values are presented as mean ± standard deviation or n (%)

### Surgical procedure

All patients underwent a preoperative urine culture 14 days prior to surgery. In cases of positive culture, targeted antibiotic therapy was administered for 5 days based on the antibiogram. Additionally, perioperative antibiotic prophylaxis was systematically administered at the time of anesthetic induction according to institutional protocols. For the purposes of the present analysis, the preoperative urine culture was defined as the sample obtained before initiation of antibiotic treatment, irrespective of the subsequent microbiological clearance prior to surgery.

Flexible ureteroscopy (FURS) was performed under general anesthesia using flexible ureteroscopes with an outer diameter ranging from 6.3 to 9.5 Fr, including both reusable and single-use platforms. When deemed necessary, a ureteral access sheath (9.5–12 Fr) was inserted to facilitate repeated access, improve visibility, and optimize irrigation flow.

Irrigation was delivered using 0.9% saline solution at room temperature through a standardized gravity-based system positioned at approximately 60 cm, corresponding to an irrigation pressure of ~ 40 cmH₂O. All procedures were performed using the TraxerFlow™ dual-port gravity irrigation system, allowing controlled flow. Upon entry into the bladder and subsequently into the renal pelvis, irrigation was temporarily interrupted by manually closing the three-way valve prior to urine sampling. The system includes a dedicated third line for direct urine aspiration, thereby minimizing potential dilution during sample collection.

Stone fragmentation was performed using a controlled “painting” technique [6], with laser pulses delivered in long-pulse mode to optimize dusting efficiency and laser–stone interaction. At the conclusion of the procedure, stent placement was performed at the discretion of the operating surgeon based on intraoperative findings.

All urine samples were processed according to institutional microbiological protocols. Culture results were classified as negative, positive for a single identified pathogen, polymicrobial, or unknown when microbiological identification was inconclusive.

### Data collection and outcomes

Demographic, clinical, perioperative, microbiological, and postoperative data were collected retrospectively from institutional electronic medical records. Baseline variables included age, sex, body mass index (BMI), diabetes mellitus, arterial hypertension, history of recurrent urinary tract infection, stone morphology, preoperative ureteral stenting status, and preoperative urine culture results. Information regarding preoperative antibiotic therapy, including timing and resistance patterns, was also recorded.

All patients underwent ureteroscopy (URS) for urinary stone disease. Urine samples were obtained at three predefined timepoints: (1) bladder urine collected intraoperatively upon entry unto the bladder, (2) pelvic urine aspirated intraoperatively prior to lithotripsy, and (3) pelvic urine obtained after completion of laser lithotripsy. For each sample, standard urine dipstick analysis was performed immediately, assessing leukocytes, nitrites, protein, pH, blood, specific gravity, ketones, and glucose. In parallel, urine culture (ECBU) was obtained according to institutional microbiological protocols.

Dipstick positivity for infection was evaluated using two predefined criteria: (1) an “OR” definition (presence of leukocytes and/or nitrites), and (2) an “AND” definition (presence of both leukocytes and nitrites). These definitions were selected a priori to evaluate the impact of diagnostic thresholds on performance characteristics.

The primary outcome was microbiologically confirmed urine culture positivity at each sampling site. The secondary outcome was severe postoperative infection. Infectious complications were defined as any clinically relevant infection developing during the postoperative course, including febrile episodes requiring antimicrobial treatment, sepsis, or septic shock. Severe infections were defined as cases of sepsis or septic shock in accordance with international definitions [7].

All postoperative infectious complications were identified through clinical documentation and laboratory findings during hospitalization and follow-up. Missing data were not imputed, and analyses were performed using available-case methodology.

### Statistical analysis

All statistical analyses were performed using the R software environment for statistical computing and graphics (version 4.5.1; http://www.r-project.org/). The primary endpoint was microbiologically confirmed urine culture positivity (ECBU). The diagnostic performance of urine dipstick testing was evaluated separately for bladder urine, intraoperative pelvic urine, and post-laser pelvic urine samples. Dipstick positivity was defined using two predefined criteria: (1) the OR definition, considered positive if leukocytes and/or nitrites were present; and (2) the AND definition, considered positive only if both leukocytes and nitrites were present.

For each sampling timepoint, 2 × 2 contingency tables were constructed to compare dipstick results with corresponding urine culture results. Diagnostic performance metrics, including sensitivity, specificity, positive predictive value (PPV), and negative predictive value (NPV), were calculated. 95% confidence intervals (CIs) were estimated using the Wilson score method. In addition to timepoint-specific analyses, a pooled descriptive analysis was conducted by aggregating all paired dipstick–culture samples across timepoints. Comparisons between the OR and AND definitions were primarily descriptive, with ROC analysis providing additional quantitative assessment of discriminative performance.

In addition, receiver operating characteristic (ROC) curve analysis was performed to further evaluate the diagnostic performance of urine dipstick testing for predicting culture positivity at each sampling site. The area under the curve (AUC) with 95% confidence intervals was calculated for each sampling site and for each dipstick definition, including the predefined OR (leukocytes and/or nitrites) and AND (leukocytes and nitrites) criteria, as well as the composite dipstick score. In addition to the predefined OR and AND definitions, a composite dipstick score was constructed by summing leukocyte and nitrite positivity, yielding an ordinal variable ranging from 0 to 2. ROC analyses were conducted using the pROC package in R.

A sensitivity analysis was performed excluding patients with positive preoperative urine cultures who received targeted antibiotic therapy, to assess the robustness of dipstick diagnostic performance.

## Results

### Study population and sample distribution

A total of 238 patients undergoing flexible ureteroscopy were included in the analysis (Table 1). Across the three predefined sampling timepoints, 634 paired dipstick–culture urine samples were available, including 217 bladder samples, 210 intraoperative pelvic samples, and 207 post-laser pelvic samples. Overall, culture positivity was observed in 128 of 634 samples (20.2%). Culture positivity rates by sampling site are detailed in (Table 2).

**Table 2:** Diagnostic performance of urine dipstick testing for predicting urine culture positivity across sampling sites

Timepoint	Total Samples	Culture Positive	Culture Negative	Positivity Rate
Bladder urine	217	40	177	18.4 (13.8–24.1)
Pelvic urine (intraoperative)	210	48	162	22.9 (17.7–29)
Pelvic urine (post-laser)	207	40	167	19.3 (14.5–25.2)
Pooled (all samples)	634	128	506	20.2 (17.2–23.5)

^a^ Values are presented as sensitivity, specificity, positive predictive value (PPV), and negative predictive value (NPV) with 95% confidence intervals

^b^ OR definition: leukocytes and/or nitrites

^c^ AND definition: leukocytes and nitrites

### Diagnostic performance — OR definition (Leukocytes and/or Nitrites)

Using the OR definition, urine dipstick testing demonstrated high sensitivity and negative predictive value across all sampling sites, although specificity and positive predictive value remained limited (Table 3). Diagnostic performance was most balanced for intraoperative pre-laser pelvic urine samples, whereas post-laser samples demonstrated lower sensitivity but higher specificity.

**Table 3:** Diagnostic performance of urine dipstick positivity (leukocytes and/or nitrites) for predicting culture positivity by sampling site and pooled analysis

Sample / Timepoint	*N* (paired samples)	Sensitivity	Specificity	PPV	NPV
Bladder urine	217	85% [70.9–92.9]^*^	36.7% [30–44]	23.3% [17.2–30.8]	91.5% [82.8–96.1]
Pelvic urine (intraoperative)	210	85.4% [72.8–92.8]	45.1% [37.6–52.7]	31.5% [24.2–40]	91.2% [83–95.7]
Pelvic urine (post-laser)	207	70% [54.6–81.9]	61.7% [54.1–68.7]	30.4% [22–40.5]	89.6% [82.6–93.9]
Pooled (all samples)	634	80.5% [72.8–86.4]	47.6% [43.3–52]	28% [23.6–32.8]	90.6% [86.5–93.6]

^a^ Dipstick positivity defined as leukocytes and/or nitrites. Values are % with 95% confidence intervals (Wilson method). Culture positivity refers to the corresponding urine culture at the same sampling site

**Fig. 1:**
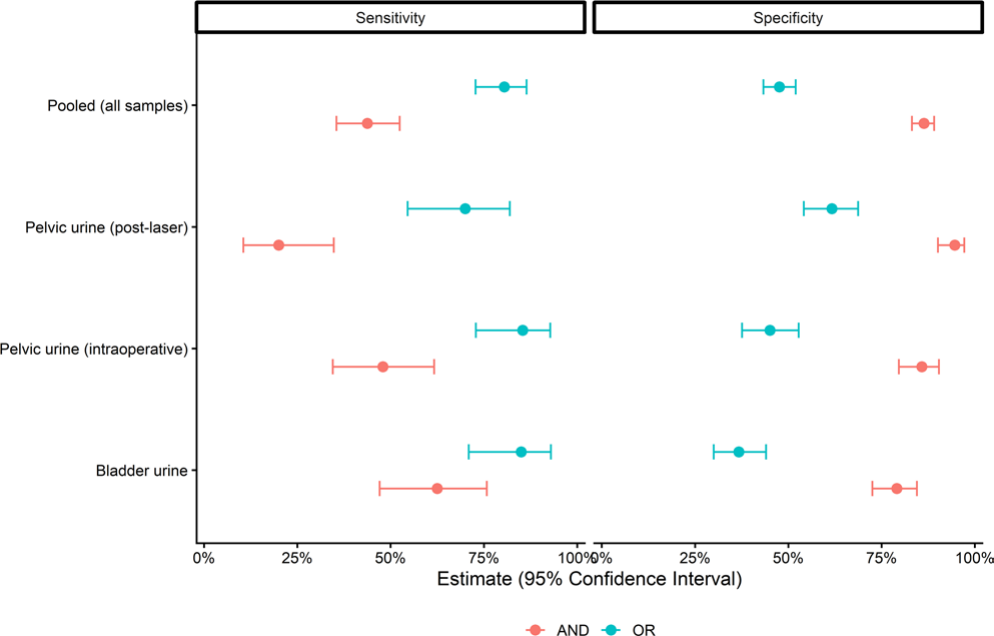
Forest plot of sensitivity and specificity with 95% confidence intervals for urine dipstick positivity predicting culture positivity across sampling sites and pooled analysis. Dipstick positivity was defined as leukocytes and/or nitrites (OR) or leukocytes and nitrites (AND). Confidence intervals were calculated using the Wilson method

In the pooled analysis (*n* = 634), the OR definition yielded a sensitivity of 80.5% (95% CI 72.8–86.4), specificity of 47.6% (95% CI 43.3–52.0), PPV of 28.0% (95% CI 23.6–32.8), and NPV of 90.6% (95% CI 86.5–93.6). Predictive values and culture positivity prevalence across sampling sites are illustrated in (Fig. 1).

### Diagnostic performance — AND definition (Leukocytes and Nitrites)

Results for the AND definition are presented in (Table 4). Compared with the OR definition, requiring the simultaneous presence of leukocytes and nitrites reduced sensitivity while substantially improving specificity and positive predictive value across all sampling sites. The highest specificity was observed in post-laser pelvic urine samples.

**Table 4:** Diagnostic performance of urine dipstick positivity (leukocytes and nitrites) for predicting culture positivity across sampling sites

Sample / Timepoint	*N* (paired samples)	Sensitivity	Specificity	PPV	NPV
Bladder urine	217	62.5% [47–75.8]^*^	79.1% [72.5–84.4]	40.3% [29–52.7]	90.3% [84.6–94]
Pelvic urine (intraoperative)	210	47.9% [34.5–61.7]	85.8% [79.6–90.3]	50% [36.1–63.9]	84.8% [78.5–89.5]
Pelvic urine (post-laser)	207	20% [10.5–34.8]	94.6% [90.1–97.1]	47.1% [26.2–69]	83.2% [77.2–87.8]
Pooled (all samples)	634	43.8% [35.5–52.4]	86.4% [83.1–89.1]	44.8% [36.4–53.5]	85.9% [82.6–88.6]

^a^ Dipstick positivity defined as leukocytes and nitrites

^b^ Values are % with 95% confidence intervals (Wilson method)

^c^ Culture positivity refers to the corresponding urine culture at the same sampling site

**Fig. 2:**
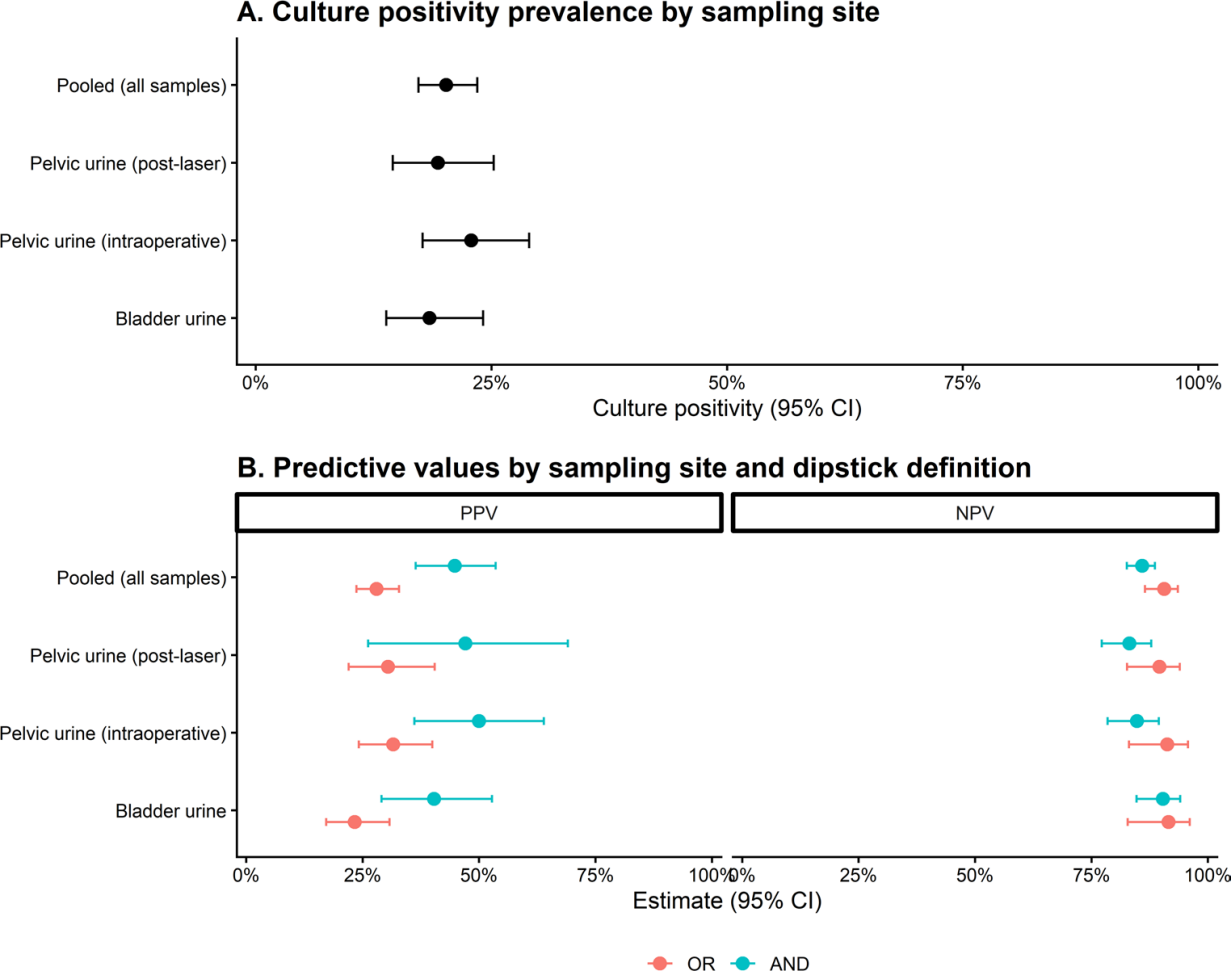
**a**,** b** Culture positivity prevalence and predictive values of urine dipstick across sampling sites. (**a**) Prevalence of culture positivity for bladder, intraoperative pelvic, post-laser pelvic, and pooled samples. (**b**) Positive predictive value (PPV) and negative predictive value (NPV) of dipstick positivity by sampling site using OR (leukocytes and/or nitrites) and AND (leukocytes and nitrites) definitions. Error bars indicate 95% confidence intervals (Wilson method)

In the pooled analysis (*n* = 634), the AND definition yielded a sensitivity of 43.8% (95% CI 35.5–52.4), specificity of 86.4% (95% CI 83.1–89.1), PPV of 44.8% (95% CI 36.4–53.5), and NPV of 85.9% (95% CI 82.6–88.6). The trade-off between sensitivity and specificity across sampling sites and positivity definitions is illustrated in (Fig. 2a and b).

### Receiver operating characteristic (ROC) analysis — culture positivity

**Fig. 3:**
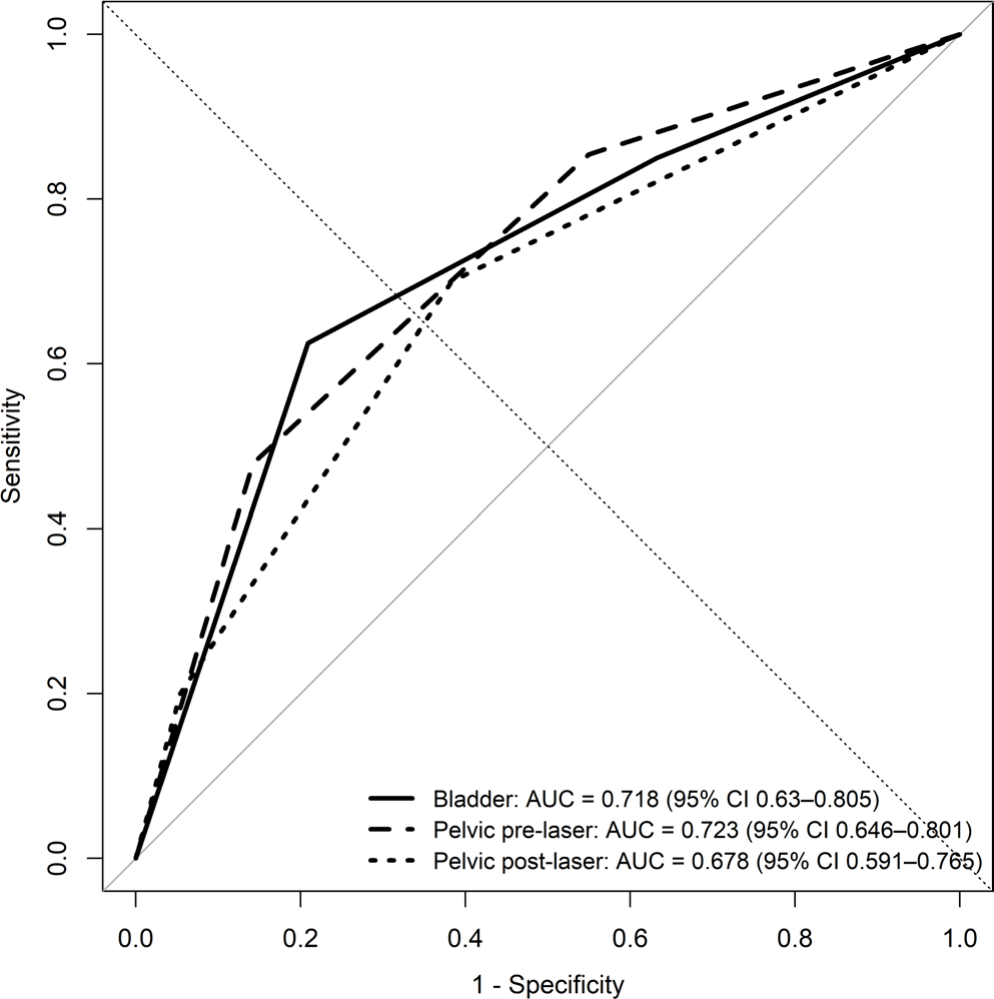
Receiver operating characteristic curves for dipstick score (0–2) predicting urine culture positivity at bladder, pelvic pre-laser, and pelvic post-laser sampling sites. The x-axis represents 1 − specificity and the y-axis represents sensitivity. The diagonal dashed line indicates no discriminative ability. AUC values with 95% confidence intervals are shown in the legend

ROC curve analysis demonstrated moderate diagnostic performance of urine dipstick testing for predicting culture positivity across sampling sites. The composite dipstick score showed the highest discriminative performance and is presented in (Fig. 3), while AUC values with 95% confidence intervals for the OR, AND, and score definitions are reported in (Supplementary Figure S2). The highest discriminative ability was observed for pelvic urine sampled prior to laser fragmentation, with an AUC of 0.723 (95% CI 0.646–0.801) using the composite dipstick score. Bladder dipstick testing showed similar performance (AUC 0.718, 95% CI 0.630–0.805), while post-laser pelvic samples demonstrated slightly lower discrimination (AUC 0.678, 95% CI 0.591–0.765). Across all sampling sites, the composite dipstick score consistently outperformed both OR and AND definitions. The AND definition showed improved specificity but reduced overall discriminative performance in certain settings, particularly in post-laser samples.

### Severe postoperative infection

Severe postoperative infection, defined as sepsis or septic shock, occurred in 10 of 238 patients (4.2%, 95% CI 2.3–7.5%). An exploratory analysis assessed the association between urine dipstick positivity and severe postoperative infection. Using the OR definition (leukocytes and/or nitrites), pooled dipstick testing showed a sensitivity of 30% and a negative predictive value (NPV) of 93.8% for severe infection. No additional statistical modeling was performed due to the limited number of severe infection events.

### Sensitivity analysis excluding patients with preoperative culture positivity

A sensitivity analysis excluding patients with positive preoperative urine cultures who received targeted antibiotic therapy (*n* = 85) was performed, resulting in a restricted cohort of 153 patients. In this analysis, the negative predictive value of intraoperative dipstick testing remained high and was slightly increased across all sampling sites (bladder: 97.9% vs. 91.5%; pre-laser pelvic: 96.7% vs. 91.2%; post-laser: 96.2% vs. 89.6%). Positive predictive values decreased, consistent with the lower prevalence of culture positivity in this subgroup. Overall, diagnostic performance patterns remained consistent with the primary analysis (Supplementary Table S1, Supplementary Figures S1–S2).

## Discussion

In this retrospective, single-center study of 238 patients undergoing URS, we evaluated the diagnostic performance of intraoperative urine dipstick testing across multiple sampling sites for predicting microbiologically confirmed culture positivity. Culture positivity ranged from 18.4% to 22.9%, consistent with previously reported rates in ureteroscopy populations [2, 4]. Overall, dipstick testing demonstrated moderate diagnostic performance, with the OR definition (leukocytes and/or nitrites) providing higher sensitivity and the AND definition offering improved specificity, reflecting the expected trade-off between these approaches [8].

Diagnostic performance was characterized by relatively high negative predictive values and lower positive predictive values across sampling sites. Prior antibiotic treatment and perioperative prophylaxis may have reduced bacterial load and contributed to false-negative cultures, potentially explaining the relatively low positive predictive values observed [4, 9]. Importantly, despite standardized preoperative culture-guided antibiotic therapy and perioperative prophylaxis, severe infectious complications still occurred in a subset of patients [2, 4]. This suggests that microbiological eradication may be incomplete or that clinically relevant infection risk persists despite appropriate management. In this context, urine dipstick markers may reflect inflammatory or residual infectious processes not fully captured by culture alone, supporting their potential role as complementary tools rather than replacements for microbiological testing.

Diagnostic performance varied by sampling site, with intraoperative pelvic urine obtained prior to laser fragmentation demonstrating the most balanced metrics and highest discriminative performance. This likely reflects more accurate representation of upper tract microbial burden before procedural manipulation [10]. In contrast, post-laser samples showed reduced performance, likely influenced by irrigation, hematuria, and tissue disruption. These findings highlight the importance of sampling timing and suggest that pre-laser pelvic urine provides the most clinically informative intraoperative assessment.

Receiver operating characteristic analysis further supported these findings, demonstrating moderate discriminative performance overall. The composite dipstick score yielded the highest AUC values, particularly for pre-laser pelvic samples, reinforcing the added value of integrating leukocyte and nitrite information into an ordinal framework rather than relying solely on binary definitions.

The clinical applicability of these findings lies in perioperative risk stratification rather than direct decision-making. The clinical value of intraoperative dipstick testing does not lie in confirming infection, but in providing immediate negative-risk information before culture results become available. Intraoperative dipstick testing should be interpreted as an adjunctive tool, providing real-time information that may complement preoperative assessment. A negative dipstick result, particularly in pre-laser pelvic samples, may support a lower likelihood of culture positivity, whereas a positive result should prompt increased clinical vigilance rather than immediate therapeutic escalation. In such cases, clinicians may consider closer postoperative monitoring, early microbiological confirmation, and individualized antibiotic strategies in higher-risk patients. However, given the moderate diagnostic performance observed, dipstick findings should always be interpreted in conjunction with clinical assessment and established perioperative protocols [11].

These findings are consistent with recent observational evidence supporting the prognostic value of intraoperative urine sampling as shown by Nannola et al [12]. Furthermore, they provide a foundation for future prospective validation studies. If confirmed, improved intraoperative risk stratification may help refine procedural decision-making, including operative time considerations and postoperative management strategies such as inpatient observation versus same-day discharge.

In exploratory analysis, dipstick testing demonstrated limited utility for predicting severe postoperative infection. Although the negative predictive value was high, this is largely attributable to the low prevalence of severe infection and does not reflect strong discriminative performance. Notably, sensitivity was low (30%), indicating that the majority of severe infection cases were not detected. These findings highlight that dipstick testing should not be used to predict severe postoperative outcomes and should be interpreted with caution in this context [13].

To address the potential confounding effect of preoperative antibiotic therapy, we performed a sensitivity analysis excluding patients with positive preoperative cultures who received targeted treatment. The persistence of high negative predictive values in this restricted cohort suggests that the rule-out performance of dipstick testing is not driven by prior antibiotic exposure. These findings support the robustness of our results and reinforce the role of intraoperative dipstick testing primarily as a tool for identifying low-risk patients.

This study has several limitations that should be acknowledged. First, its retrospective, single-center design and single-surgeon setting may limit the generalizability of the findings to other institutions and surgical practices. However, this approach ensured procedural consistency and standardized sampling, strengthening internal validity. Second, the analysis of severe infection outcomes is limited by the small number of events (*n* = 10), restricting statistical power and precluding definitive conclusions. Third, we did not adjust for potential confounders such as antibiotic exposure and resistance patterns. Finally, although measures were taken to minimize dilution during sampling, the influence of irrigation, particularly in post-laser conditions, cannot be completely excluded [4, 9].

Overall, intraoperative urine dipstick testing may offer practical value during URS, particularly given its high negative predictive value for excluding culture positivity and severe infection. Integration of rapid diagnostic information could potentially support more individualized perioperative antibiotic strategies, aligning with antimicrobial stewardship principles [11].

## Conclusions

Intraoperative urine dipstick testing demonstrates moderate diagnostic performance for predicting culture positivity during ureteroscopy, with results influenced by sampling site and positivity definition. Pre-laser pelvic urine sampling provides the most informative intraoperative assessment. While dipstick testing may offer supportive real-time information for perioperative risk stratification, its performance is insufficient for standalone clinical decision-making, particularly for predicting severe infection. Further prospective studies are required to validate its role and define its integration into perioperative management strategies.

## Supplementary Information

Below is the link to the electronic supplementary material.

Supplementary Material 1

Supplementary Material 2

Supplementary Material 3

## Data Availability

The data that support the findings of this study are available from the corresponding author, Carlos Gonzalez Gonzalez, upon reasonable request.
