# Supplementary material for: Diagnostic performance of intraoperative urine dipstick testing during ureteroscopy: association with culture positivity and severe infection
Source: Urolithiasis. 2026 Jun 13;54(1):113. doi: 10.1007/s00240-026-02020-2 (PMC13264553; doi:10.1007/s00240-026-02020-2)
Supplement: Supplementary file 1 — Supplementary Material 1 [file 240_2026_2020_MOESM1_ESM.docx]

| **Cohort** | **Sampling site** | **N** | **Positive** | **Sens (%)** | **Spec (%)** | **PPV (%)** | **NPV (%)** |
| --- | --- | --- | --- | --- | --- | --- | --- |
| Overall cohort | Intraoperative Bladder | 217 | 40 | 85% | 36.7% | 23.3% | 91.5% |
|  | Pelvic pre-laser | 210 | 48 | 85.4% | 45.1% | 31.5% | 91.2% |
|  | Pelvic post-laser | 207 | 40 | 70% | 61.7% | 30.4% | 89.6% |
| Excluding preop culture-positive patients | Intraoperative Bladder | 140 | 11 | 90.9% | 36.4% | 10.9% | 97.9% |
|  | Pelvic pre-laser | 136 | 11 | 81.8% | 47.2% | 12% | 96.7% |
|  | Pelvic post-laser | 133 | 8 | 62.5% | 61.6% | 9.4% | 96.2% |

**Table S1** Diagnostic performance of intraoperative urine dipstick testing (sensitivity analysis excluding preoperative culture-positive patients)
