# Supplementary material for: Diagnostic performance of intraoperative urine dipstick testing during ureteroscopy: association with culture positivity and severe infection
Source: Urolithiasis. 2026 Jun 13;54(1):113. doi: 10.1007/s00240-026-02020-2 (PMC13264553; doi:10.1007/s00240-026-02020-2)
Supplement: Supplementary file 2 — Supplementary Material 2 [file 240_2026_2020_MOESM2_ESM.docx]

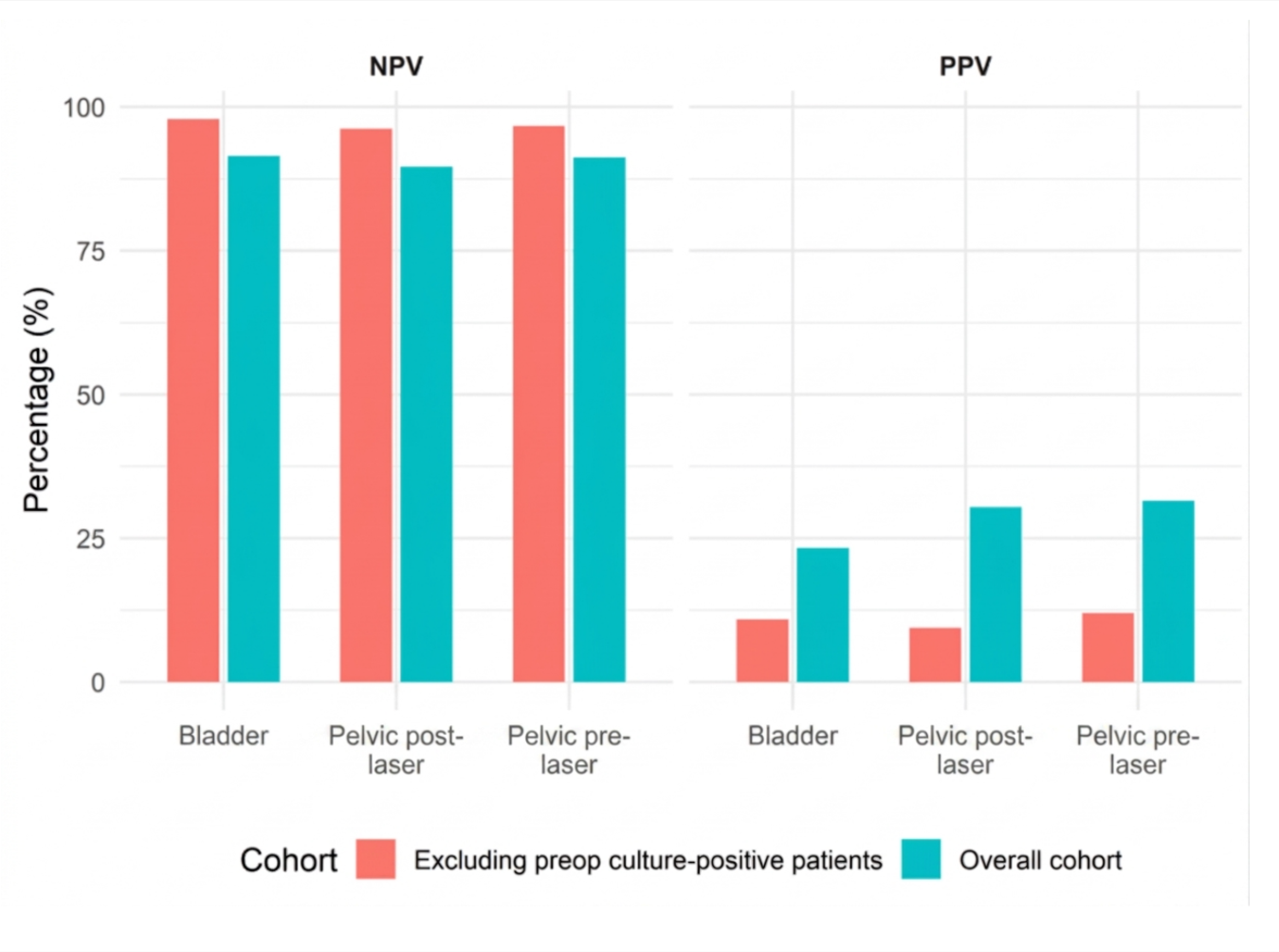


**Fig. S1** Diagnostic performance of intraoperative urine dipstick testing for prediction of culture positivity across sampling sites (bladder, pelvic pre-laser, and pelvic post-laser) in the overall cohort and after exclusion of patients with preoperative culture positivity. Bars represent positive predictive value (PPV) and negative predictive value (NPV) for each sampling site.
