# Supplementary material for: Diagnostic performance of intraoperative urine dipstick testing during ureteroscopy: association with culture positivity and severe infection
Source: Urolithiasis. 2026 Jun 13;54(1):113. doi: 10.1007/s00240-026-02020-2 (PMC13264553; doi:10.1007/s00240-026-02020-2)
Supplement: Supplementary file 3 — Supplementary Material 3 [file 240_2026_2020_MOESM3_ESM.docx]

**
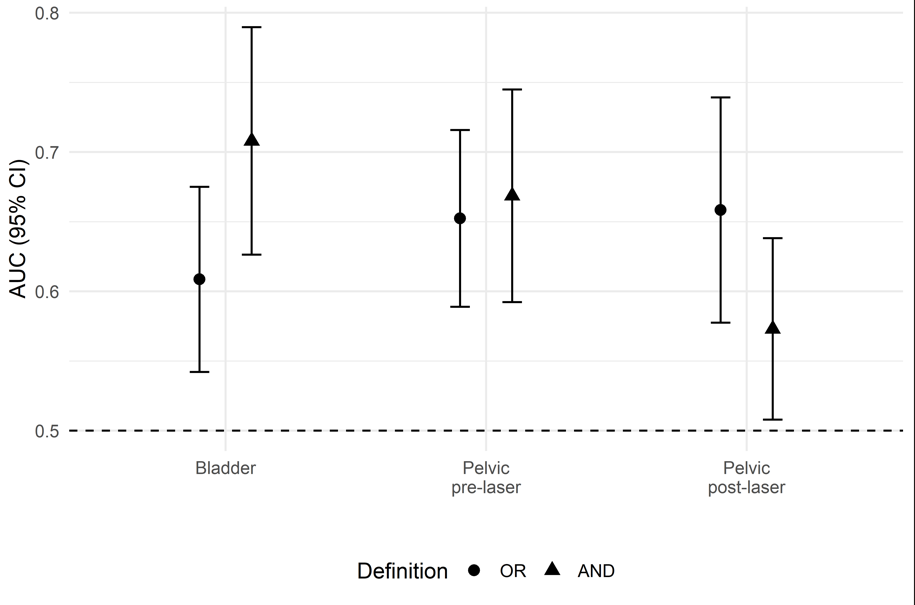
**

**Fig. S2**  Area under the curve (AUC) values with 95% confidence intervals for urine dipstick testing using OR and AND definitions across bladder, pelvic pre-laser, and pelvic post-laser sampling sites. The dashed horizontal line indicates an AUC of 0.5.
